# Pharmacokinetic and Safety Evaluation of Various Oral Doses of a Novel 1:20 THC:CBD *Cannabis* Herbal Extract in Dogs

**DOI:** 10.3389/fvets.2020.583404

**Published:** 2020-09-29

**Authors:** Alan Chicoine, Kate Illing, Stephanie Vuong, K. Romany Pinto, Jane Alcorn, Kevin Cosford

**Affiliations:** ^1^Department of Veterinary Biomedical Sciences, Western College of Veterinary Medicine, University of Saskatchewan, Saskatoon, SK, Canada; ^2^College of Pharmacy and Nutrition, University of Saskatchewan, Saskatoon, SK, Canada; ^3^Department of Small Animal Clinical Sciences, Western College of Veterinary Medicine, University of Saskatchewan, Saskatoon, SK, Canada

**Keywords:** cannabinoid, cannabidiol (CBD), tetrahydrocannabinol (THC), cannabichromene (CBC), nonlinear pharmacokinetics, hyperesthesia, adverse drug events (ADE), canine (dog)

## Abstract

**Objective:** To determine the pharmacokinetics (PK) and safety of various oral doses of a *Cannabis* herbal extract (CHE) containing a 1:20 ratio of Δ9-tetrahydrocannabinol (THC):cannabidiol (CBD) in 13 healthy Beagle-cross dogs.

**Methods:** Single-dose PK was assessed after oral administration of CHE at low, medium, or high doses [2, 5, or 10 mg CBD and 0.1, 0.25, or 0.5 mg THC per kg of body weight (bw), respectively; *n* = 6 per group]. Dogs were monitored for adverse events for up to 48 h post-dose. Evaluations of neurological signs, clinical laboratory abnormalities, and other adverse events were performed in two separate study phases: a multiple-dose phase with 12 dogs receiving five medium doses (5 mg CBD/kg bw) at 12 h intervals, and a single low-dose (2 mg CBD/kg bw), randomized, blinded, negative controlled study with 13 dogs.

**Results:** Cannabinoids CBD, THC, CBC, and metabolites 6-OH-CBD, 7-OH-CBD, 11-OH-THC, and THC-COOH were quantified in plasma. CBD and THC were rapidly absorbed (mean *T*_max_ of 1.9–2.3 h) and initially depleted rapidly (mean CBD *T*_1/2β_ of 2.3–2.6 h). A prolonged elimination phase (mean CBD *T*_1/2λ_ of 13.3–24.4 h) was observed. CBD and THC concentrations increased in a dose-dependent (non-linear) manner, with disproportionally greater cannabinoid exposure relative to the dose increase. Neurological signs (hyperesthesia or proprioceptive deficits) were noted in five of six dogs in the high-dose group, but only occasionally or rarely in the medium- and low-dose groups, respectively. Presence and severity of clinical signs correlated with plasma cannabinoid concentrations. Dogs appeared to develop a tolerance to cannabinoid effects after multiple CHE doses, with fewer neurological signs noted after the final (fifth) vs. first dose. No clinically meaningful changes in blood count or chemistry values occurred after multiple CHE doses.

**Clinical Significance:** Dogs tolerated the 1:20 THC:CBD formulation well at low and medium doses, but clinically meaningful neurological signs were observed at high doses. Because of non-proportional increases in plasma cannabinoid concentrations with increasing doses, as well as potential differences in CHE product composition and bioavailability, the possibility of adverse events and dose regimen consistency should be discussed with dog owners.

## Introduction

*Cannabis*-derived herbal extracts (CHEs) are increasingly popular for both human and animal use, with recent legalization of such products in Canada and some European countries and American states. There is significant interest in evaluating CHE products for a variety of applications in both human and animal health, including chronic osteoarthritis (1, 2), epilepsy (3, 4), neuropathic pain (5), and nausea/emesis (6). Substantial differences can exist between CHE formulations produced by licensed vs. unlicensed producers, with wide variations in cannabinoid composition and label accuracy (7, 8). Despite widespread use in pet animals (9), no CHE products have been approved for veterinary use by North American regulatory authorities, and veterinarians cannot legally authorize use of CHE products in their patients.

Most available evidence of cannabinoid toxicity in veterinary species is associated with the accidental ingestion of recreational cannabinoids (10); assessment of efficacy and toxicity of various CHE formulations is limited in veterinary patients. Such CHE products contain varying amounts of cannabinoids, particularly cannabidiol (CBD) and Δ9-tetrahydrocannabinol (THC), as well as oil bases or excipients that influence cannabinoid absorption (11). Other CHE formulations used in veterinary pharmacokinetics (PK) studies include microencapsulated oil beads and transdermal creams (12). Before large-scale clinical efficacy trials of CHE products can be performed, particularly in client-owned animals, preliminary assessments of cannabinoid PK, and safety should be performed in a controlled setting. The objective of this study was to generate such data in a colony of teaching dogs after administering various doses of a CBD-enriched CHE product containing 1:20 THC:CBD.

## Materials and Methods

### Dogs

Thirteen research-bred Beagle cross dogs were used in this study. Mean age was 22.6 months (range, 22–24 months), with mean body weight of 14.9 kg (range, 11.8–18.7 kg). There were three intact females, four neutered females, two intact male, and four neutered males. Dogs were housed in individual kennels or small group runs for the duration of the study. All dogs were in good health based on history, physical examination, and complete blood count and chemistry profiles. This work was approved by the University of Saskatchewan's Animal Research Ethics Board and adhered to the Canadian Council on Animal Care guidelines for humane animal use (Animal Use Protocol No. 20190007).

### Test Item

A CBD-enriched CHE containing nominal concentrations of 20 mg CBD, 1 mg THC, and 4 mg cannabichromene (CBC) per mL was purchased from a Licensed Producer (Aurora Cannabis Inc.). Exemption for use of the CHE in animals was granted by Health Canada via an Experimental Studies Certificate and Cannabis Tracking and License System research exemption. Certificates of Analysis were provided by Aurora Cannabis Inc. for all product batches used in the study. Dose regimens for both the PK and multidose study phases were based on CBD dose regimens used in previously published studies (1, 12, 13).

### PK Study Design

The PK study was performed in two phases. In the first PK phase, 12 dogs were randomly assigned, accounting for both sex and body weight, to receive a single oral dose of CHE, with one dog remaining in reserve. Two doses were initially tested, a high [10 mg CBD + 0.5 mg THC/kg of body weight (bw)] and medium (5 mg CBD + 0.25 mg THC/kg bw) dose with *n* = 6 per dose group. In the second PK phase, occurring 8 weeks later, 6 of the 13 dogs were randomly selected and administered a low CHE dose (2 mg CBD + 0.1 mg THC/kg bw). See Table 1 for cannabinoid composition of all dose groups in the PK phases. Dogs were fasted overnight, and indwelling catheters placed in the cephalic vein prior to CHE dose administration. After the CHE was vigorously mixed by hand, the specific dose volume for each dog was drawn up in a syringe and immediately administered via inserting the syringe tip in the dog's mouth near the base of the tongue, holding the mouth closed until swallowing occurred. No regurgitation or vomiting occurred immediately after administration. Dosage volumes ranged from 1.1 mL (low-dose group) to 9.4 mL (high-dose group). Dogs were monitored intermittently for the duration of the PK phase (24–48 h), and any adverse events were recorded.

**Table 1:** Cannabinoid doses administered to dogs via a 1:20 THC:CBD *Cannabis* herbal extract.

**CHE dose group**	**mg CBD/ kg bw**	**mg THC/ kg bw**	**mg CBC/ kg bw**
Low	2	0.1	0.4
Medium	5	0.25	1.0
High	10	0.5	2.0

Blood for cannabinoid analysis was collected from all dogs at the following times after CHE administration: 0 (pre-treatment), 0.5, 1.0, 1.5, 2.0, 3.0, 4.0, 6.0, 8.0, 12.0, 24.0, and 48.0 h. Dogs in the low-dose group were only sampled up to 24 h post-dose because of limited time of animal availability. All blood samples were collected via indwelling catheters unless the catheter became dislodged or blocked, at which point samples were collected via jugular venipuncture. Blood (3.0 mL) was collected into lithium heparin tubes and immediately placed on ice. To determine pre-CHE treatment baseline values, a portion of the time 0 h blood sample from all dogs in the first PK phase was submitted for complete blood count and clinical chemistry analysis at a commercial laboratory (Prairie Diagnostic Services, Saskatoon, Saskatchewan, Canada). All blood samples were centrifuged within 2 h of collection at 1,200 × *g* for 10 min. Plasma was then harvested in 200 μL aliquots in Eppendorf Protein Lo-bind microcentrifuge tubes and frozen at −80°C. Samples were stored for no longer than 194 days before analysis.

### Multidose Study

Immediately following the 48 h blood collection, the same 12 dogs used in the PK study phase were administered five consecutive CHE doses at 12 h intervals. Both medium- and high-dose groups were originally planned for the multidose study phase, but because of adverse events noted in five of six dogs in the high-dose group during the first PK phase, the medium dose (5 mg CBD + 0.25 mg THC/kg bw) was used for all 12 dogs. Dogs were not fasted during the multidose portion and had free access to their allotment of food. Blood was collected by jugular venipuncture 2 h following the final CHE dose for post-treatment complete blood count and clinical chemistry analysis (Prairie Diagnostic Services), as well as plasma cannabinoid analysis to assess potential cannabinoid accumulation. Blood samples were processed and analyzed as per the PK study portion.

### Adverse Event and Neurological Assessment

During the PK study portions, dogs were evaluated in individual kennels for coughing, vomiting, salivation, tremors, head bobbing, general mentation, or any other abnormalities readily apparent upon routine handling. Dogs were directly observed over an 8 h period post-dosing.

During the multiple-dose study phase, neurological examinations were performed (KRP, KC) on each dog at multiple time points following the first and fifth (final) CHE doses. For each dog, the same examiner performed all examinations each day. Dogs were examined immediately prior to CHE administration (baseline) and at approximately 2 and 6 h post-treatment. The neurologic assessment consisted of a qualitative checklist for each of the following: attitude, behavior, mentation, proprioception (ability to correct knuckling), balance (response to gentle pressure on the hip), gait, urinary incontinence, pupillary light reflex, and pupil size (normal, constricted, or dilated). Kennel staff recorded any evidence of gastrointestinal disturbances (e.g., vomit, diarrhea) in the kennels or other adverse events during the multidose phase.

### Low-Dose Neurological Assessment

Following the final (24 h) blood sample of the low-dose PK study phase, an assessment of potential adverse events and neurological signs using the low CHE dose was performed. Ten dogs were randomly selected to receive the low CHE dose (non-fasting), with the remaining three dogs left as untreated controls. Neurological examinations were performed on treated and untreated dogs as per the multidose study phase (pre-dose and at ~2 and 6 h post-dose). The examiner (K.R.P.) was blinded to the treatment status of the dogs.

### Plasma Sample Preparation and Liquid Chromatography–Tandem Mass Spectrometry Analysis

The analytical method validated for cannabinoid analysis in dog plasma was a modified version of a previously validated assay for human plasma (14). Full validation parameters are presented in Table 2. Analytical standards of CBD, THC, CBC, 11-OH-THC, and THC-COOH (1 mg/mL dissolved in methanol) and the deuterated internal standards, CBD-d3, THC-d3, 11-OH-THC-d3, and THC-COOH-d3 (0.1 mg/mL in methanol), were purchased from Cerilliant (Round Rock, TX, USA). CBC-d9 (0.1 mg/mL in methanol) was purchased from Cayman Chemical (Ann Arbor, MI, USA). 6-OH-CBD, 7-OH-CBD, and 7-OH-CBD-d9 (0.5 mg) were purchased from Toronto Research Chemicals (Toronto, Ontario, Canada) and dissolved in methanol at a concentration of 1 mg/mL. All analytical standards were stored at −20°C. Liquid chromatography–mass spectrometry (LC-MS)–grade methanol, water, acetonitrile, formic acid, and ammonium formate were purchased from Thermo Scientific (Waltham, MA, USA).

**Table 2:** Method validation data for canine plasma LC-MS/MS assay.

	**CBD**	**6-OH-CBD**	**7-OH-CBD**	**THC**	**11-OH-THC**	**THC-COOH**	**CBC**
Internal standard	CBD-D3	7-OH-CBD-D9	7-OH-CBD-D9	THC-D3	11-OH-THC-D3	THC-COOH-D3	CBC-D9
Standard curve (ng/mL)	1.97–250	1.97–250	3.91–250	1.97–250	1.97–250	1.97–250	1.97–250
QC range (ng/mL)	5–175	5–175	10–175	5–175	5–175	5–175	5–175
LLOQ (ng/mL)	1.97	1.97	3.91	1.97	1.97	1.97	1.97
LOD (ng/mL)	0.98	0.98	0.98	0.98	0.98	0.98	0.98
Average cannabinoidrecovery (%)	91.15	79.43	87.11	95.24	92.51	93.60	86.62
Intraday accuracy (%)	LQC = 113.4 MQC = 103.5 HQC = 103.8	LQC = 107.7 MQC = 106.9 HQC = 103.5	LQC = 107.5 MQC = 103.2 HQC = 99.9	LQC = 113.5 MQC = 103.7 HQC = 105.6	LQC = 109.8 MQC = 106.8 HQC = 105.4	LQC = 112.9 MQC = 105.2 HQC = 104.2	LQC = 113.5 MQC = 105.7 HQC = 106.6
Intraday precision (%)	LQC = 0.32 MQC = 3.8 HQC = 2.1	LQC = 4.7 MQC = 3.6 HQC = 1.7	LQC = 3.2 MQC = 1.7 HQC = 1.4	LQC = 2.1 MQC = 2.4 HQC = 1.0	LQC = 5.5 MQC = 3.3 HQC = 2.6	LQC = 1.1 MQC = 3.8 HQC = 1.5	LQC = 1.2 MQC = 4.4 HQC = 2.5
Interday accuracy (%)	LQC = 113.8 MQC = 103.9 HQC = 104.0	LQC = 108.1 MQC = 104.2 HQC = 100.4	LQC = 104.5 MQC = 102.6 HQC = 99.0	LQC = 111.4 MQC = 102.3 HQC = 103.1	LQC = 110.0 MQC = 104.0 HQC = 104.3	LQC = 113.7 MQC = 103.6 HQC = 103.1	LQC = 113.1 MQC = 103.9 HQC = 104.1
Interday precision (%)	LQC = 0.56 MQC = 4.5 HQC = 2.7	LQC = 2.9 MQC = 2.7 HQC = 2.6	LQC = 4.6 MQC = 2.2 HQC = 2.4	LQC = 2.7 MQC = 2.9 HQC = 1.8	LQC = 4.5 MQC = 4.2 HQC = 2.7	LQC = 1.9 MQC = 3.1 HQC = 2.2	LQC = 1.1 MQC = 2.9 HQC = 1.7

*LQC, low-quality control (5 ng/mL for CBD, CBC, 11-OH-THC, THC-COOH, and 6-OH-CBD; 10 ng/mL for THC and 7-OH-CBD); MQC, medium-quality control (100 ng/mL); HQC, high-quality control (175 ng/mL)*.

LC-MS/MS analysis was conducted on an Agilent 1290 binary pump LC system (Agilent Technologies Canada, Mississauga, Ontario, Canada) equipped with an online degasser, connected to ABSciex 6500 QTrap mass spectrometer with Turbo Spray (ABSciex, Concord, Ontario, Canada). Cannabinoids were chromatographically separated on an Agilent Zorbax Eclipse Plus Phenyl Hexyl column (4.6 × 100 mm, 5 μm), set at a column temperature of 50°C. The mobile phase consisted of water with 0.1 mM ammonium formate and 0.1% formic acid (mobile phase A) and methanol with 0.1 mM ammonium formate and 0.1% formic acid (mobile phase B). Mobile phase was delivered at a flow rate of 0.5 mL/min under gradient conditions as follows: 73% B over 0–0.5 min, 73–77% B over 0.5–7.0 min, 77–85% B over 7–7.5 min, 85% B from 7.5 to 13.5 min, and then returning to 73% B over 13.6–17.5 min. The autosampler was set to 4°C, and the injection volume was 5 μL.

Triple quadrupole MS acquisition was conducted using electrospray in positive ionization mode, with an ion spray voltage of 5,500 V and source temperature of 600°C. Collision-activated dissociation using nitrogen gas was set to 10 V. Curtain gas, nebulizer gas, and heater gas were set to 50, 70, and 60 psi, respectively. Multiple reaction monitoring (MRM) transitions were utilized for quantitative analysis. ABSciex Analyst 1.6.2 and 1.7 were used for data acquisition. ABSciex MultiQuant 3.0.1 was used for data analysis.

Working standards were prepared by serial dilution using methanol, with concentrations ranging from 39.06 to 5,000 ng/mL for CBD, CBC, 11-OH-THC, THC-COOH, and 6-OH-CBD and 78.13–5,000 ng/mL for THC and 7-OH-CBD. Quality control solutions were prepared similarly, with concentrations of 100 ng/mL [low-quality control (LQC) for CBD, CBC, 11-OH-THC, THC-COOH, and 6-OH-CBD], 200 ng/mL (LQC for THC and 7-OH-CBD), 2,000 ng/mL [medium-quality control (MQC)], and 3,500 ng/mL [high-quality control (HQC)]. Internal standards, CBD-d3, THC-d3, CBC-d9, THC-COOH-d3, 11-OH-THC-d3, 7-OH-CBD-d9 (100 μg/mL), were diluted in methanol to prepare 1.67 μg/mL working internal standards. All working standards were stored at −20°C until day of analysis.

Standard curves were prepared by adding 10 μL of working standard to 190 μL of blank pooled dog plasma, with final concentrations ranging from 1.97 to 250 ng/mL for THC, CBD, CBC, 11-OH-THC, THC-COOH, and 6-OH-CBD, and 3.91–250 ng/mL for 7-OH-CBD. Samples with CBD concentrations exceeding the upper limit of quantification were diluted with blank dog plasma spiked with internal standard, using dilution factors of 10× or 100×, to fit CBD concentrations within the standard curve. Low-, medium-, and high-quality control samples (LQC, MQC, and HQC, respectively) followed the same procedure, with final concentrations of 5 ng/mL (LQC for CBD, CBC, 11-OH-THC, THC-COOH, and 6-OH-CBD), 10 ng/mL (LQC for THC and 7-OH-CBD), 100 ng/mL (MQC), and 175 ng/mL (HQC). Sample processing consisted of the addition of 610 μL of cold acetonitrile and internal standard (10 μL IS: 600 μL ACN) to 200 μL of dog plasma for protein precipitation. Samples were vortex mixed for 30 s and centrifuged at 14,000 rpm for 10 min at 4°C. Supernatant was filtered through Agilent Captiva EMR-Lipid 96-well plates under vacuum; 500 μL of filtrate was transferred to amber high-performance liquid chromatography vials and injected into the LC-MS/MS instrument.

### PK Analysis

The plasma cannabinoid concentration vs. time (C-T) data for each dog were analyzed by non-compartmental techniques using GraphPad Prism 8. Final PK parameters were expressed as mean ± SD. The maximum plasma concentration (*C*_max_) and time to *C*_max_ (*T*_max_) were determined from visual inspection of the C-T curves. The log-linear terminal rate constant, λ_z_, was estimated as the terminal slope [i.e., last 3 points (12–48 h)] of the natural logarithmic C-T curve using linear regression analysis, while the half-life was estimated as the ratio 0.693/k. However, a β-phase rate constant was determined from *T*_max_ to 12 h. The area under the C-T curve from 0 to 12 h after dosing (AUC_0−12h_) was determined using the linear trapezoidal rule. The total area under the curve extrapolated to infinity (AUC_0−inf_) was calculated by adding the *C*_lastobs_/λ_z_ + AUC_0−last_. The β-phase half-life (*T*_1/2, β_) and terminal λ_z_ half-life (*T*_1/2_, _λ*z*_) were calculated as ln2/β and ln2/λ_z_, respectively. To facilitate parameter comparisons between dose groups, *C*_max_ and AUC_0−12h_ were subsequently dose-normalized (divided by dose administered). Non-compartmental analysis also produced estimated apparent volume of distribution (*V*_d_/*F*) and apparent clearance (Cl_S_/F).

### Statistical Analysis

Baseline and post-treatment complete blood count and chemistry results were analyzed using paired *t*-test or Wilcoxon rank sum tests (normal and non-normally distributed data, respectively). Dose-normalized *C*_max_ and AUC_0−12h_ data for CBD and THC were analyzed using one-way analysis of variance, with *post-hoc* comparison of groups using Tukey least significant difference. To evaluate possible accumulation, 2 h concentrations after single-dose and post-final dose study portions (5 mg CBD/kg dose group only) were compared using paired *t*-test. For all statistical analyses, *p* < 0.05 was defined as the cutoff for statistical significance. Because of limited numbers of observations and variation in assessment procedures between dose groups, statistical evaluation of the neurological evaluations and adverse events was considered inappropriate; only incidence of findings is reported.

## Results

### CHE Formulations Used

The CHE batches used in these studies contained 19.7–19.9 mg CBD, 1.0–1.1 mg THC, 3.6–4.3 mg CBC, and 0.2 mg cannibigerol per mL. No cannabinol, elemental impurities, mycotoxins, or pesticides were detected in any CHE batches used.

### Dose Administration

Administration of CHE was well-tolerated in all dogs. Two dogs had mild cough shortly after swallowing the CHE in the PK phase, but no vomit or regurgitation was noted immediately after dosing during any study phase. Transient lip-licking was noted following CHE administration in two dogs during the PK phase and occasionally during the multidose portion, but loss of CHE was not visible at any point.

### PK Results

**Figure 1:**
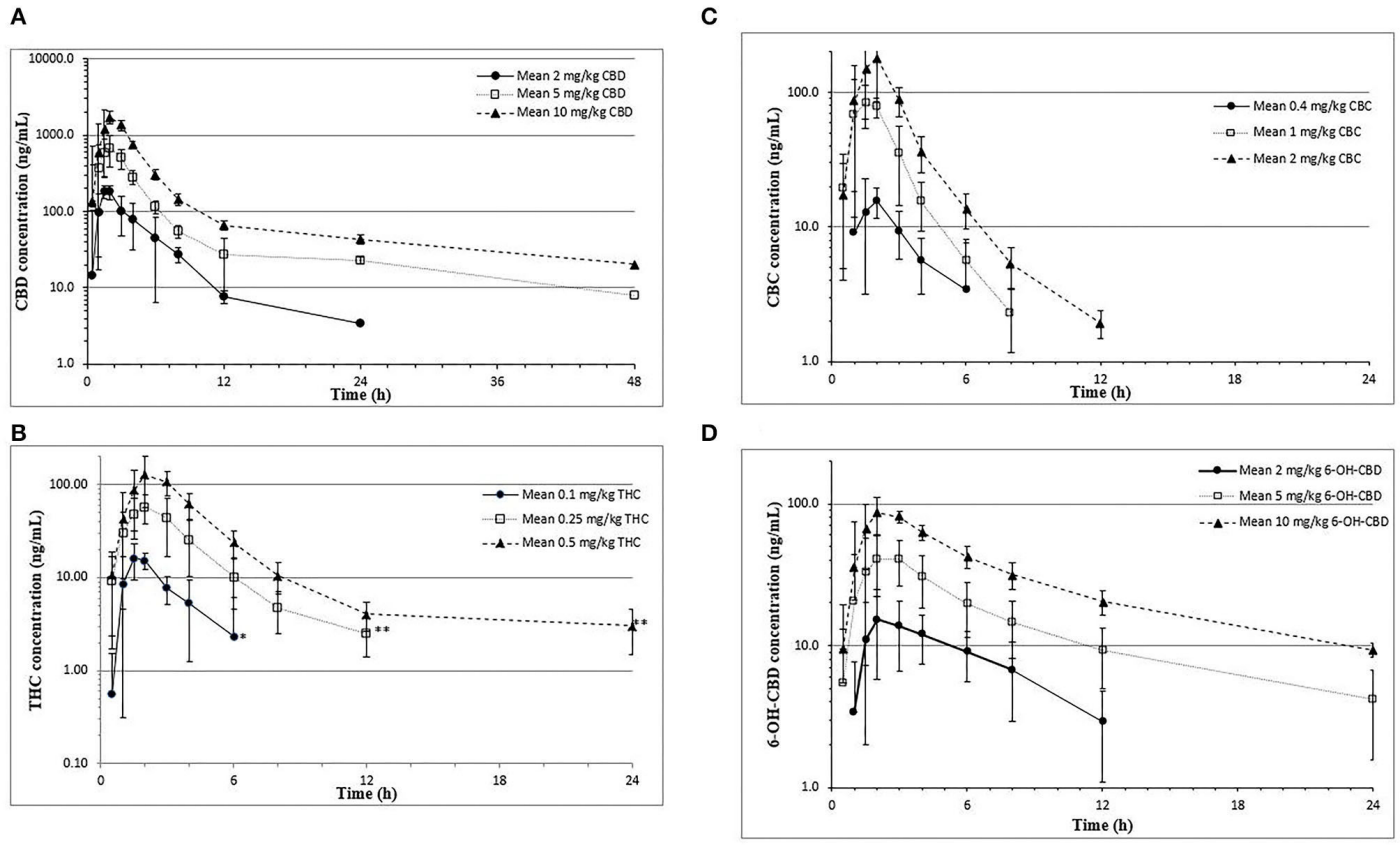
Mean ± SD plasma CBD **(A)**,THC **(B)**, CBC **(C)**, and 6-OH-CBD **(D)** concentrations over time in fasted beagle cross dogs (*n* = 6/dosing group; mixed gender) receiving a single oral dose of 1:20 THC:CBD CHE formulation at different dose sizes. ^*^Mean value based on three quantifiable concentrations. ^**^Mean value based on five quantifiable concentrations.

Mean plasma logarithmic concentration vs. time curves for all dose groups are shown in Figure 1. In the medium- and high-dose groups, CBD concentrations in plasma were quantifiable (1.97 ng/mL) up to 48 h post-dose, and these disposition curves showed a slow terminal elimination phase. The primary CBD metabolite formed was 6-OH-CBD and was quantifiable at 48 h but only in the high-dose group. Other cannabinoids (THC and CBC) and metabolites (11-OH-THC) were quantifiable for only up to 12 h. Concentrations of the metabolite, 7-OH-CBD, were intermittently quantifiable, and did not exceed 40 ng/mL. The metabolite, THC-COOH, was detectable (>0.49 ng/mL), but not quantifiable, in plasma samples from two dogs in the high-dose group, and not detected in all other samples. An unknown metabolite was also detected with the same MRM transition as CBD, THC, and CBC, eluting at 9.65 min (immediately prior to CBD).

PK parameters derived for the cannabinoids CBD, THC, CBC, and 6-OH-CBD after a single oral dose; as well as potential steady-state concentrations (blood samples taken 2 h following the final 5 mg CBD/kg bw in the multidose study), are shown in Table 3. Plasma concentrations for other cannabinoid metabolites (7-OH-CBD, 11-OH-THC, and THC-COOH) were not suitable for PK analysis due to the low number of quantifiable concentrations observed. Dose adjusted AUC_0−12h_ (i.e., AUC_0−12h_/dose) showed a statistically significant increase with increasing doses for THC (ANOVA *p* = 0.026). Apparent volume of distribution (*V*_d_/*F*) and apparent clearance (Cl_S_/*F*) were calculated but not reported as the variance was extremely high, and bioavailability (*F*) was unknown.

**Table 3:** Mean (SD) cannabinoid PK parameters in fasted beagle cross dogs (*n* = 6/dosing group; mixed gender) receiving a single oral dose of 1:20 THC:CBD CHE formulation at different dose sizes.

**Cannabinoid**	**Dose (mg/kg)**	***T*_**max**_ (h)**	***C*_**max**_ (ng/mL)**	**Dose-Adjusted *C*_**max**_ (ng/mL per mg/kg)^1^**	***T*_**1/2β**_ (h)^2^**	AUC_**0–12h**_ (ng * h/mL)	Dose-Adjusted AUC_**0–12h**_ (ng * h/mL per mg/kg)^1^	**T_**1/2λ**_ (h)^3^**	AUC_**last**_ (ng * h/mL)	**“Steady-state” conc. (ng/mL)^4^**
CBD	2	2.1 (1.0)	213 (49)	107^A^ (24)	2.5 (0.5)	692 (292)	346^A^ (146)	13.3 (4.1)	759 (335)	NA
	5	1.9 (0.6)	838 (304)	168^A^ (61)	2.6 (0.4)	2,433 (911)	487^A^ (182)	23.1 (8.2)	2,935 (1,244)	1,264 (579)
	10	2.3 (0.5)	1,868 (698)	187^A^ (70)	2.3 (0.2)	5,883 (2,181)	588^A^ (218)	24.4 (9.5)	7,239 (2,393)	NA
THC	0.1	2.1 (1.0)	17.5 (5.5)	175^A^ (55.4)	1.6 (1.5)	43.5 (16.0)	434.7^A^ (160)	NA	NA	NA
	0.25	2.1 (0.5)	67.6 (20.3)	270.2^A^ (81.3)	1.9 (0.5)	203.9 (73.3)	815.5^AB^ (293.1)	NA	NA	79.4 (35.4)
	0.5	2.3 (0.5)	138.3 (56.4)	276.6^A^ (112.8)	1.9 (0.2)	451.5 (178.8)	903.0^B^ (357.7)	NA	NA	NA
CBC	0.4	2.5 (1.7)	17.7 (5.4)	44.3 (13.4)	2.1 (2.2)	53.0 (26.1)	132.4 (65.3)	NA	NA	NA
	1.0	1.8 (0.8)	100.9 (30.3)	100.9 (30.3)	1.6 (0.4)	220.3 (52.2)	220.3 (52.2)	NA	NA	69.0 (34.5)
	2.0	2.3 (0.6)	191.6 (87.0)	95.8 (43.5)	1.9 (0.2)	449.4 (160.8)	224.7 (80.4)	NA	NA	NA
6-OH-CBD	2 (CBD)	3.4 (2.1)	16.9 (8.2)	8.5 (4.1)	3.2 (0.7)	94.5 (38.4)	47.3 (19.2)	NA	NA	NA
	5 (CBD)	2.2 (0.7)	47.7 (18.1)	9.5 (3.6)	4.7 (0.9)	247.8 (99.4)	49.6 (19.9)	NA	NA	41.7 (13.2)
	10 (CBD)	2.3 (0.6)	94.9 (17.0)	9.5 (1.7)	5.1 (0.7)	516.7 (62.6)	51.7 (6.3)	NA	NA	NA

*T_max_, time to maximum concentration; C_max_, maximum concentration; AUC, area under the plasma concentration vs. time curve; T_1/2_, half−life; NA, notapplicable*.

*CBD and THC statistical analysis: Differing alphabetical superscripts in each column indicate statistically significant (p < 0.05) differences between mean values (ANOVA with post-hoc Tukey LSD)*.

1 *Dose-adjusted value (parameter value divided by mg/kg dose)*.

2 *T_1/2β_ phase, from 4 to 12 h post-dose*.

3 *T_1/2λ_ (terminal elimination) phase, from 12 to 48 h post-dose*.

4 *Taken 2 h after five consecutive CHE doses at 12 h intervals (n = 12), true steady-state concentrations. Unlikely reached at this time*.

### Adverse Events Observed in the PK Phase

Although specific neurological examinations were not conducted during the PK collection periods, neurological signs were apparent during routine handling and blood collection in five of six dogs in the high-dose group (Table 4). No dog demonstrated obvious cannabinoid “intoxication” (i.e., altered mentation, sedation), but hyperesthesia (overreaction to normal auditory/visual/tactile stimuli) and proprioceptive deficits were noted. Signs typically were first observed within 1–2 h of dosing and subsided within 4–6 h post-dose. Other adverse events noted in the high-dose group included ptyalism (1/6 dogs), urinary incontinence (1/6 dogs), and small amounts of vomit (3/6 dogs). The only adverse event noted in the medium-dose group during the PK phase was vomit (1/6 dogs) and cough (1/6 dogs). No dogs in the low CHE dose group (n = 6) displayed adverse events during the PK study phase.

**Table 4:** Adverse events observed in beagle cross dogs (*n* = 6/dosing group; mixed gender) receiving a single oral dose of 1:20 THC:CBD CHE formulation at different dose sizes.

**Dosage (mg/kg)**		**n/Group**	**Number of dogs exhibiting adverse event (% of group)**							
**CBD**	**THC**		**Head bobbing**	**Torso swaying (at rest)**	**Hyperesthesia (audio, visual, or tactile stimuli)**	**Ataxia**	**Urinary incontinence**	Cough^*^	**Ptyalism**	Vomit^**^
2	0.10	6	0	0	0	0	0	0	0	0
5	0.25	6	0	0	0	0	0	1 (17)	0	1 (17)
10	0.5	6	1 (17)	2 (33)	3 (50)	1 (17)	1 (17)	1 (17)	1 (17)	3 (50)

* *Occurring immediately post-dosing*.

** *Occurring at any point up to 24 h post-dose*.

### Adverse Events Observed During Multiple-Dose and Low-Dose Phases

Given that adverse events were predominantly exhibited in the high-dose (but not medium-dose) groups in the first PK phase, all dogs in subsequent study phases were administered the medium or low CHE doses. Detailed neurological examination findings after both single-dose (negative control, low, and medium dose) and multiple-dose (medium) regimens are shown in Table 5. Neurological changes (including pupil dilation, mild ataxia, noise sensitivity, and various delayed reflex responses) could be detected at 2 h after administration of the single 5 mg CBD/kg bw dose, but signs were significantly diminished or absent within 6 h. Despite specifically focusing on neurological signs via performance of neurological examinations by an experienced examiner, the signs observed were substantially less pronounced than the conspicuous signs noted in the high-dose group in the previous PK phase. After the final dose in the multiple-dose phase (fifth consecutive dose of 5 mg CBD/kg bw at 12 h intervals), substantially fewer neurological signs were observed than after the single 5 mg CBD/kg bw dose (Table 5). After a single oral dose of 0 or 2 mg CBD/kg bw, neurological signs, and other adverse events were infrequent and minor. The evaluator blinded to treatment groups could confidently identify only one dog as being CHE-treated based on neurological signs; the other 12 dogs could not be distinguished as CHE-treated or negative controls.

**Table 5:** Neurological and other adverse events observed in beagle cross dogs (mixed gender) receiving single or multiple oral doses of 1:20 THC:CBD CHE formulation at different dose sizes.

**Dosing regimen**	**Dosage (mg/kg)**		**n/ Group**	**Number of dogs exhibiting adverse event (% of group)**								
	**CBD**	**THC**		**Mydriasis**	**Miosis**	Sluggish PLR^*^	**Ataxia**	**Delayed hopping**	**Delayed knuckling**	**Noise sensitivity**	**Urinary incontinence**	**Vomit**
Single	0	0	3	0	2 (67)	0	0	0	0	0	0	0
	2	0.1	10	0	5 (50)	0	2 (20)	1 (10)	0	0	0	2 (20)
	5	0.25	12	9 (75)	0	0	3 (25)	6 (50)	2 (17)	6 (50)	1 (8)	0
Multiple	5	0.25	12	3 (25)	0	0	0	4 (33)	0	0	0	0

* *Pupillary light reflex*.

### Blood Count and Chemistry Panel Results

Selected complete blood count/chemistry values obtained from blood samples taken pre-treatment and after the final dose in the multiple-dose phase (fifth consecutive dose of 5 mg CBD/kg bw at 12 h intervals) are shown in Table 6. Overall, pre-dose, and post-dose values for all parameters were consistently within reference ranges, although the mean differences in pre-dose and post-dose values were statistically significantly different for some parameters (*p* < 0.05; paired *t*-test or Wilcoxon rank sum).

**Table 6:** Selected mean hematology and clinical chemistry parameters in beagle cross dogs (mixed gender) taken pre-CHE treatment and post-fifth medium CHE dose (5 mg CBD/kg bw).

**Test**	**Units**	**Reference**	**Mean pre-treatment**	**Mean post-treatment**	**Mean change**	**% Difference**
Sodium	mmol/L	140–153	147.1	146.3	−0.8	−1
Potassium	mmol/L	3.8–5.6	4.5	4.5	0.0	1
Na:K ratio		28–38	33.1	32.5	−0.6	−2
Chloride	mmol/L	105–120	110.4	108.8	−1.6	−1
Bicarbonate	mmol/L	15–25	20.5	18.4	−2.1^*^	−10
Anion gap	mmol/L	12–26	20.7	23.7	3.0^**^	15
Calcium	mmol/L	1.91–3.03	2.6	2.6	0.0	0
Phosphorus	mmol/L	0.63–2.41	1.2	1.5	0.3^*^	23
Magnesium	mmol/L	0.70–1.16	0.9	0.9	0.0	1
Urea	mmol/L	3.5–11.4	4.5	5.0	0.5^*^	12
Creatinine	μmol/L	41–121	74.3	72.4	−1.8	−2
Amylase	U/L	343–1,375	707.1	710.2	3.1	0
Lipase	U/L	25–353	86.2	81.0	−5.2	−6
Glucose	mmol/L	3.1–6.3	5.1	5.2	0.2	3
Cholesterol	mmol/L	2.70–5.94	5.0	4.8	−0.1	−2
Total bilirubin	μmol/L	1.0–4.0	1.7	1.2	−0.6^**^	−33
Direct bilirubin	μmol/L	0–2	0.8	0.6	−0.1^**^	−18
Indirect bilirubin	μmol/L	0–2.5	1.0	0.5	−0.4^**^	−46
Alkaline phosphatase	U/L	9–90	51.3	56.9	5.7	11
γ-Glutamyl transferase	U/L	0–8	4.9	2.8	−2.1^**^	−42
Alanine aminotransferase	U/L	19–59	46.2	41.3	−4.9	−11
Glutamate dehydrogenase	U/L	0–7	3.3	3.4	0.2	5
Creatinine kinase	U/L	51–418	126.8	98.3	−28.4^**^	−22
Total protein	g/L	55–71	58.5	56.2	−2.3	−4
Albumin	g/L	32–42	36.7	36.1	−0.6	−2
Globulin	g/L	20–34	21.8	20.1	−1.8	−8
A:G ratio		1.06–1.82	1.7	1.8	0.1	7
Leukocytes	×10^9^/L	4.9–15.4	7.9	8.0	0.1	2
Segmented neutrophils	×10^9^/L	3.0–10.0	4.7	4.9	0.2	5
Eosinophils	×10^9^/L	0–1.1	0.7	0.6	−0.2	−22
Lymphocytes	×10^9^/L	1.2–5.0	2.0	2.1	0.1	4
Monocytes	×10^9^/L	0.08–1.0	0.5	0.5	0.0	−3
Erythrocytes	×10^12^/L	5.8–8.5	7.3	6.4	−0.9	−12

* *p < 0.05, paired t-test (normal distribution)*.

** *p < 0.05, Wilcoxon rank sum test (non-normal distribution)*.

## Discussion

The PK of CBD and/or THC after administration of various CHE formulations and dose regimens have recently been published in dogs (1, 12, 13, 15–17). The range of CBD and THC doses previously reported guided the dose range selected for this trial (2–10 mg CBD and 0.1–0.5 mg THC per kg bw), with the exception of those studies using much higher THC doses (16, 17).

The disposition of cannabinoids in canine plasma in this study was generally comparable to previously published results. Absorption after oral administration was rapid, with mean *T*_max_ of approximately 2 h for CBD and THC in all dose groups. The initial rate of depletion in plasma from *T*_max_ until 12 h post-dose (β-phase) was comparable for all cannabinoids, with mean β-phase half-life (*T*_1/2β_) of approximately 2 h for CBD, THC, and CBC. These values are similar to previously published results for mean CBD elimination half-life, reported between 1 and 4 h (1, 12, 13).

However, following the rapid decline in plasma CBD concentrations from 2 to 12 h, a prolonged elimination phase (λ) was observed in all dogs demonstrating quantifiable cannabinoid levels at 48 h (medium- and high-dose groups, Figure 1). The terminal elimination half-life (*T*_1/2λ_), derived from plasma samples taken between 12 and 48 h post-dose, ranged from 12 to 24 h, depending on dose group (Table 3). Published human trials have demonstrated comparably prolonged cannabinoid elimination half-lives from plasma beyond 24 h post-dose, presumably due to redistribution of cannabinoids from adipose tissue (18, 19). Previously cited studies in dogs collected plasma samples no longer than 24 h and thus failed to report a prolonged terminal phase in their cannabinoid concentration–time profiles. The data in the current study suggest accumulation of cannabinoids in a deep tissue compartment with protracted equilibration times and time to steady state within such deep tissues. This represents a potential concern for toxicity, particularly with long-term dose regimens, as concentrations may continue to accumulate within these tissues to possible toxic levels depending on the dosing regimen. In the 12 dogs administered five consecutive CHE doses at 12 h intervals, the mean plasma concentrations at 2 h post-final dose (1,264 ± 579 ng/mL) were not statistically significantly different than the mean *C*_max_ (838 ± 304 ng/mL) of the six dogs administered the same 5 mg/kg dose during the initial PK phase (*p* = 0.11; independent *t*-test). However, because of the prolonged terminal phase (terminal elimination half-life of 12–24 h), steady-state plasma concentrations were unlikely reached following five consecutive twice-daily doses. Furthermore, logistical considerations did not allow for fasting of the dogs prior to CHE administration during the multidose study portion. Dosing during a potential fed state may confound the *C*_max_ because of the effects of food on cannabinoid bioavailability (discussed below). Accumulation of cannabinoids therefore cannot be ruled out after longer durations of CHE administration in dogs and has recently been demonstrated with a pharmaceutical CBD formulation (Sativex) in dogs (20).

The mean CBD *C*_max_ values (213 ng/mL) for the low-dose group were within the range of values (102–301 ng/mL) cited from other studies, which used a 2 mg/kg dose (1, 13). However, of particular interest for veterinarians is the apparent dose-dependent (i.e., non-linear) increase in cannabinoid plasma concentrations with increasing CHE doses. The dose-normalized plasma THC *C*_max_ was statistically significantly higher for the high-dose group when compared to the low-dose group. The CBD *C*_max_, and AUC_0−12h_ values for both THC and CBD also show a clear (if not statistically significant) trend toward disproportionally greater cannabinoid exposures relative to the dose increase. Similar results were observed in another dog trial using 2–8 mg CBD/kg bw dose range (1) and in one out of seven pediatric human patient administered similar doses of a comparable 1:20 THC:CBD CHE product as used in this study (3). Conversely, non-linear kinetics were not apparent in a different canine study using higher (roughly 10–20 mg/kg bw) CBD doses (12) or in model simulations based on data from trials using various CBD formulations in humans (21). Possible physiological explanations for potential non-linear kinetics may be enzyme (cytochrome P450) saturation at higher cannabinoid doses. Saturation of P450 enzymes in the intestinal mucosa or hepatocytes could lead to increased oral bioavailability (3, 19). Because of the increased risk of adverse events at higher doses (especially if exhibiting non-linear kinetics), veterinarians should caution clients who choose to increase CHE doses administered to their pets, as for many unapproved CHE formulations dosing is performed via “trial and error” approaches (7, 9).

The metabolic fate of cannabinoids in dogs has been previously reported (22), although metabolites have not been identified or quantified in most recent CHE PK studies in dogs (1, 12, 13, 15, 16). To the authors' knowledge, this is the first study to report the concurrent disposition of the cannabinoids CBD, THC, and CBC in dogs. It also confirms the production of the metabolites 6-OH-CBD, 7-OH-CBD, 11-OH-THC, and THC-COOH in dogs, although only 6-OH-CBD exposure was comparable to that of parent cannabinoids. The increased production of 6-OH-CBD (compared with 7-OH-CBD) differentiates CBD metabolism between dogs and humans, where 7-OH-CBD is the primary metabolite (19, 23, 24). Cannabinoid metabolites identified in this study, with the exception of THC-COOH, have been shown to have varying levels of affinity for CB1 and CB2 receptors and are thus presumed to have some degree of pharmacological activity (24, 25). However, the specific pharmacological activities of individual cannabinoid metabolites have not been quantified in dogs. One prominent CBD metabolite produced in both humans and dogs is 7-carboxy-cannabidiol (7-COOH-CBD), but was not included in the analytical method because of its purportedly limited pharmacological activity (17, 23, 26). However, should this metabolite be demonstrated to have pharmacological activity in dogs, its inclusion in cannabinoid assays would be warranted in future trials. An additional, unconfirmed cannabinoid substance was also detected, eluting immediately prior to CBD and with the same MRM transition as parent cannabinoids (*m/z* ratio = 315). Cannabinoids with the same molecular weight as CBD, THC, and CBC include cannabicyclol (CBL) and cannabicitran. CBL is considered the most likely candidate, as it is a degradation product of CBC, which was present in the CHE at 4.0 mg/mL. However, the identity of the unknown compound cannot be confirmed without nuclear magnetic resonance spectroscopy.

Of potential clinical relevance is the apparently higher relative bioavailability and dose-normalized exposure for THC compared to CBD. Dose-normalized mean *C*_max_ and AUC_0−12h_ values for THC were approximately 25–68% higher than the comparable values for CBD (Table 3). Because of the potential for dose-dependent (non-linear) kinetics for CBD and/or THC, and the low ratio (1:20) of THC:CBD in the CHE tested, no firm conclusions regarding comparative THC/CBD bioavailability in dogs can be made from this study. However, the potential for increased THC exposure relative to CBD is particularly concerning as some “gray market” CHE products marketed for human or veterinary use contain significantly higher quantities of THC than indicated on the label (7, 8).

Direct comparisons of relative oral bioavailability in dogs after administration of different CHE formulations between different studies are not appropriate. This is due to numerous differences in study design, which may impact assessment of oral cannabinoid bioavailability, including signalment of study populations (age/sex/breed), blood sampling schedules, and sensitivity of analytical methods used. The feeding status of animals is likely to be particularly important, as administration of lipophilic CHE formulations to humans in the fed state (particularly with high-fat meals) results in significantly higher CBD bioavailability than during fasting conditions (23, 27, 28). Conversely, a high-THC formulation had lower bioavailability when administered in the fed vs. fasting state in a small number of dogs (16). If owners choose to administer CHE products to their dogs, prudent veterinary recommendations should include consistent administration of the CHE relative to a meal (fed or fasting). Consistent administration will minimize the potential for large changes in cannabinoid exposure with repeated dosing, thus minimizing possible inefficacy or toxicity due to decreased or increased exposure, respectively.

Interestingly, 5 of the 18 individual concentration–time curves (four in the medium-dose group and one in the high-dose group) had prominent increases in CBD and THC plasma concentrations between 12 and 24 h (“secondary peaks”). The potential for improper sample labeling was investigated but ruled out because of otherwise consistent trends in plasma concentrations. The secondary plasma concentration peaks may be explained by delayed redistribution of the cannabinoids into plasma. Enterohepatic recycling of cannabinoids has been infrequently reported in humans (29), but not dogs. This phenomenon could lead to secondary cannabinoid peaks due to reabsorption after initial excretion in the bile. However, the excretion of intact parent CBD and THC or glucuronic acid conjugates of these cannabinoids in bile is likely minimal due to extensive P450 enzyme-mediated hepatic metabolism (19), and secondary peaks at 24 h were not obvious for the cannabinoid metabolites. The occurrence of secondary peaks in primarily the medium-dose but not low or high-dose groups is also puzzling and not suggestive of enterohepatic recycling. Alternatively, coprophagia may explain the secondary plasma concentration peak. The colony dogs utilized in this study were previously observed to practice coprophagia during the evening. Although coprophagy cannot be conclusively proven during this study, reingestion of previously unabsorbed parent cannabinoids from fecal material would explain the secondary peaks. The timing of the secondary peaks also fits this hypothesis, because fecal ingestion would have occurred only when the dogs were not observed by the investigators (overnight, between the 12 and 24 h sample collections). Regardless of the potential cause of the secondary peaks, any concentration–time curves demonstrating this phenomenon were analyzed only up to 12 h post-dose.

The most striking clinical finding observed was the appearance of obvious neurological signs in five of the six dogs in the high CHE dose group. None of the dogs during the study appeared to suffer from cannabinoid intoxication (changes in mentation such as stupor or depression), but hyperesthesia was readily apparent in three of six dogs in the high-dose group. This was manifested as overreaction to common stimuli, such as being startled by normal hand movements, touch, or noise. Ataxia or swaying was another obvious clinical sign in the high-dose group. Signs were apparent within 1–2 h of CHE administration and typically lasted until 4–6 h post-dose. Upon analysis of plasma cannabinoid concentrations, the clinical signs appear to highly correlate with plasma concentrations. One dog in the high-dose group, not exhibiting neurologic signs, had peak plasma CBD and THC concentrations (892 and 61.9 ng/mL, respectively) that were substantially lower than the mean *C*_max_ values for the high-dose group (1,868 and 138.3 ng/mL, respectively). The dog with the most subjectively obvious neurologic signs also had the highest CBD and THC exposure (*C*_max_ of 2,789 and 209.6 ng/mL, respectively).

Because of neurological signs observed in the high-dose group during the first PK phase, the medium dose (5 mg CBD + 0.25 mg THC per kg bw) was used for all dogs enrolled in the multidose (every 12 h × five doses) portion of the study. Detailed neurological assessments were performed pre-treatment and at 2 and 6 h post-dose, during the first and final (fifth) CHE doses. Mentation did not change in any of the dogs; all were bright, alert, and responsive. Neurological signs were noted primarily at 2 h after the first CHE dose (Table 5), but were subjectively less obvious or clinically significant than those occurring in the high CHE dose group in the PK phase. Hyperesthesia was again observed (primarily increased sensitivity to ambient noise), but in a lower proportion of dogs (*n* = 6/12) than observed in the previous high-dose phase (*n* = 5/6). Other signs, such as delayed hopping or knuckling reflexes, were only apparent because neurological examinations were specifically performed. Mydriasis was noted in 9 of the 12 dogs but was likely due to ambient light conditions. Subsequent neurologic assessments after low-dose CHE administration resulted in an opposite response (subtle miosis). Pupillary light reflexes were normal in all cases.

Interestingly, when performing the same neurological examination procedures at 0, 2, and 6 h after the final (fifth) CHE dose, the clinical signs were again less obvious than after the single (first) dose of the multidose phase administered 48 h earlier (Table 5). All dogs remained bright, alert, and responsive; signs of hyperesthesia were not evident. A delayed hopping response was again noted in two dogs. Subjectively, the dogs appeared to have developed some habituation or “tolerance” to the cannabinoid neurological (primarily hyperesthesia) effects. Such tolerance to neurological and other physiological effects is well-documented after chronic cannabis administration in humans (30), but substantially more research using longer durations of CHE administration is required to confirm a tolerance effect in dogs.

To further evaluate the potential dose-dependent nature of CHE adverse events in dogs and to determine a potential threshold for such effects, neurological examinations were again performed after single low-dose CHE administration (2 mg CBD + 0.1 mg THC/kg bw) in 10 dogs, with 3 negative controls. A comparison of pre-dose and post-dose neurological examination results indicated that neurological signs continued to decrease in incidence and significance compared to those observed in the previous study phases using the medium and high doses. Most relevant, of the 13 dogs evaluated (10 CHE-treated and 3 negative controls), the assessor blinded to treatment groups was confident in predicting only one of the dogs as “CHE-treated.”

Data in humans suggest the neurological signs in the dogs are attributable to effects of THC and not CBD. The intoxication effect of THC is mediated through CB1 receptor agonism of the endocannabinoid system, whereas CBD has minimal intoxication effect due to minimal CB1 receptor agonism (19). The interplay between CBD and THC contained in CHE products may be highly clinically relevant. On the one hand, CBD may be a partial antagonist for THC at CB1 receptors, thus minimizing its effect. Conversely, combinations of cannabinoids in herbal extracts have been reported to have an “entourage effect,” whereby CBD may exhibit synergism with other cannabinoids and/or terpenes (31, 32). This has been attributed to a decreased rate of THC metabolism through CBD-mediated P450 enzyme inhibition (33), although other mechanisms may be possible such as increased THC distribution into the central nervous system via a CBD-mediated inhibition of efflux transporter function (34, 35). It should be clarified that the current evidence regarding the potential “entourage effect” after CHE administration is based on studies in laboratory animals and humans and has not been conclusively demonstrated in dogs.

Notably, recent CHE studies in dogs generally fail to report neurological effects such as those observed in this study, despite using comparable CHE dose regimens. Some of these studies utilized CHE formulations with no or little THC and thus would be unlikely to elicit neurological changes. The current study was specifically designed to evaluate subtle neurological changes, with examinations performed by clinicians highly experienced in neurologic assessments, which might explain the absence of reporting of neurological signs in published studies. A safety-specific CHE study in dogs (17) demonstrated no adverse events utilizing CBD-only doses up to ~62 mg/kg, but increasing and medically significant neurologic adverse events with escalating doses of THC (up to 49 mg/kg) or CBD+THC (up to 12 + 8 mg/kg, respectively). Based on the extremely high THC doses used in that study (up to 98× the maximum THC dose used in this study), even more frequent and severe adverse effects would be expected. Another study (16) utilizing a relatively high THC dose in dogs (1.5 mg/kg, 3× the maximum THC dose in this study) did not mention adverse events. However, potentially low oral bioavailability of the various CHE formulations used in these studies could account for the relative lack of adverse events noted. For example, the studies noted a mean THC *C*_max_ of only 69.8 ng/mL after administering a THC dose of ~37 mg/kg to three dogs (17) and a median THC *C*_max_ of up to 24.3 ng/mL after administration of 1.5 mg THC/kg bw (16). These values are substantially lower than the mean THC *C*_max_ (138.3 ng/mL) in the highest-dose group (only 0.5 mg THC/kg bw) in the current study. It is imperative that veterinarians educate clients about the potential for substantial differences in bioavailability between CHE products, and therefore the dose used for one CHE formulation is not interchangeable with others. The potential exists for significant differences in clinical effect when using the same dose of different CHE products; i.e., the same dose empirically required for efficacy with one product may produce significant toxicity if used for another with significantly higher oral bioavailability.

Other adverse events noted during the various study phases included occasional instances of vomiting and rare episodes of urinary incontinence. These events did not correlate with dose of CHE administered, and attribution to CHE administration cannot be confirmed or denied. Laboratory testing (complete blood count and chemistry profile) was performed before the initial CHE dose in the PK phase, and after the final dose of the multidose phase 5 days later. Although some chemistry parameters were statistically significantly different following the final CHE dose, values were within reference ranges. The changes in chemistry parameter values were not considered clinically relevant and were likely attributable to changes in feeding or hydration status of the dogs at the time of blood sampling. Lack of changes in hematology or clinical chemistry parameters has been noted in other studies after chronic CHE administration in dogs (13, 17). Because of the small sample size and limited duration of CHE administration in this study, uncommon adverse events or biochemical abnormalities may not have been observed. Further studies with larger sample sizes and longer treatment duration are required to more accurately characterize the potential adverse events after administration of this CHE.

The consensus of the study authors is that the limited incidence and severity of adverse events observed in the low and medium CHE dose groups (2–5 mg CBD + 0.1–0.25 mg THC/kg bw) would be considered an acceptable risk by most dog owners considering CHE administration, particularly if “tolerance” develops after multiple CHE doses. However, the neurological signs observed in the high-dose (10 mg CBD−0.5 mg THC/kg bw) group were far more readily apparent and are less likely to be considered acceptable by owners. Veterinarians should counsel owners electing to administer CHE products of the potential for such adverse events, particularly at higher doses.

## Data Availability Statement

The datasets presented in this article are not readily available because Raw data is not available upon request, but collated data may be available for non-commercial purposes. Requests to access the datasets should be directed to Alan Chicoine, al.chicoine@usask.ca.

## Ethics Statement

The animal study was reviewed and approved by University of Saskatchewan's Animal Research Ethics Board, adhering to the Canadian Council on Animal Care guidelines for humane animal use (Animal Use Protocol Number 20190007).

## Author Contributions

AC conceived the study design, participated in the PK and safety evaluation phases, and performed the PK and statistical evaluations. KC conceived the study design, participated in the PK and safety evaluation phases, and analyzed safety data. KI participated in the PK and safety evaluation phases. SV performed analytical method validation and analyzed canine plasma samples. KP participated in the safety evaluation phase. JA provided guidance on analytical method and PK evaluation. All authors participated in writing and editing the manuscript.

## Conflict of Interest

The authors declare that the research was conducted in the absence of any commercial or financial relationships that could be construed as a potential conflict of interest.
